# Supplementary figures and images for: Progestogens for maintenance tocolysis in symptomatic women. A systematic review and meta-analysis
Source: PLoS One. 2023 Feb 22;18(2):e0277563. doi: 10.1371/journal.pone.0277563 (PMC9946203; doi:10.1371/journal.pone.0277563)

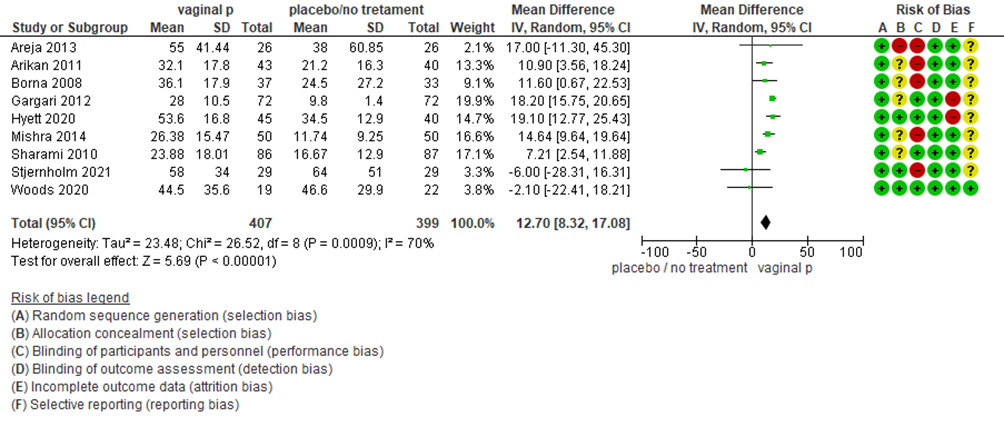

Supplement: S1 Fig — CI, confidence interval; Vaginal P, vaginal progesterone; SD, Standard deviation. (TIF) [file pone.0277563.s001.tif]

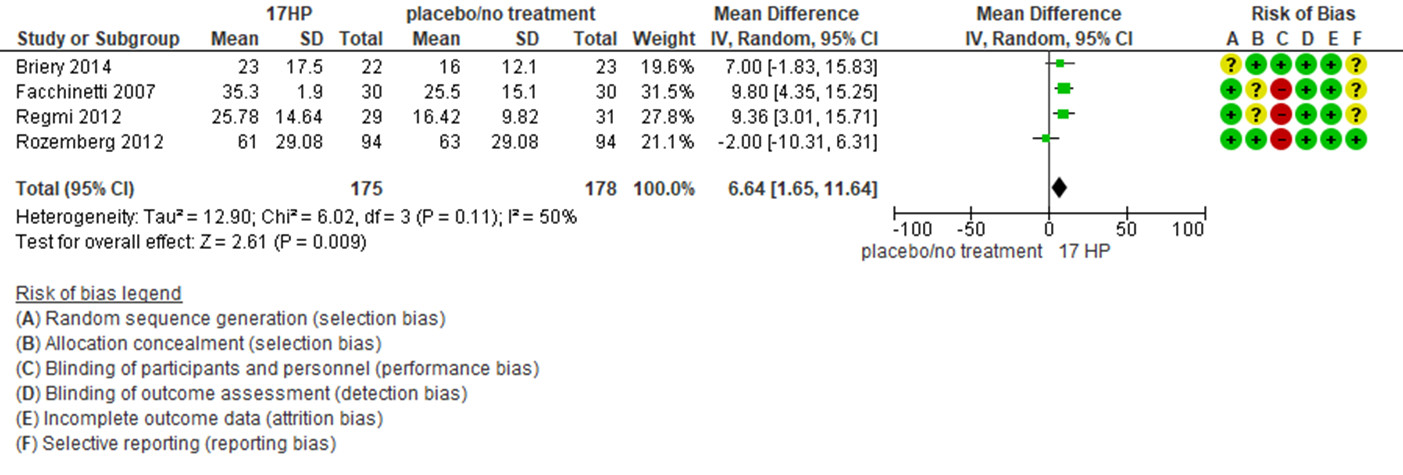

Supplement: S2 Fig — CI, confidence interval; 17-HP, 17-alfa hydroxyprogesterone; SD, Standard deviation. (TIF) [file pone.0277563.s002.tif]

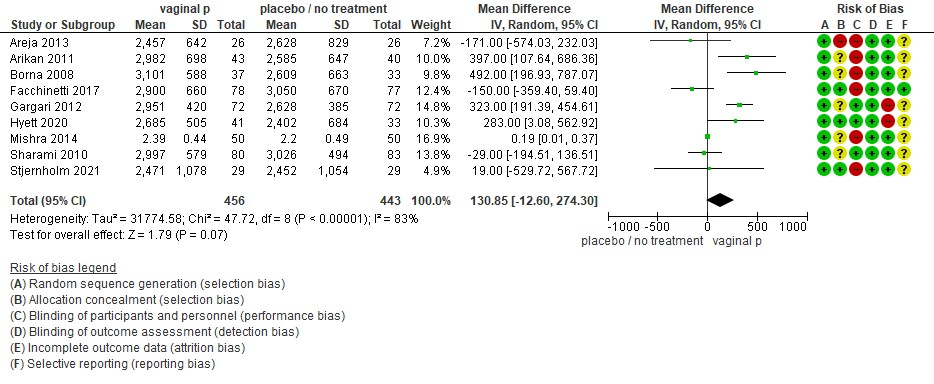

Supplement: S3 Fig — CI, confidence interval; Vaginal P, vaginal progesterone; SD, Standard deviation. (TIF) [file pone.0277563.s003.tif]

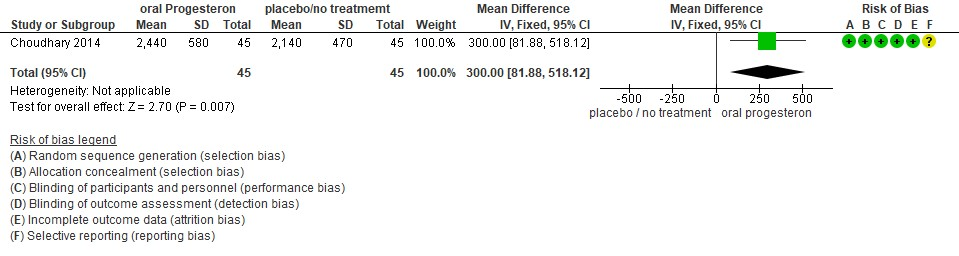

Supplement: S4 Fig — CI, confidence interval; Oral P, oral progesterone; SD, Standard deviation. (TIF) [file pone.0277563.s004.tif]

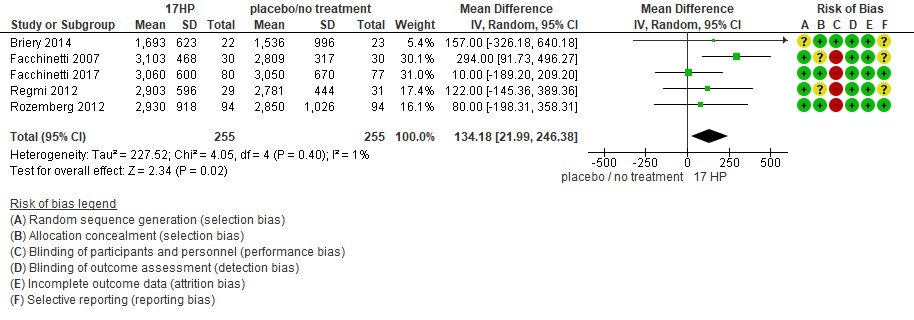

Supplement: S5 Fig — CI, confidence interval; 17-HP, 17-alfa hydroxyprogesterone; SD, Standard deviation. (TIF) [file pone.0277563.s005.tif]

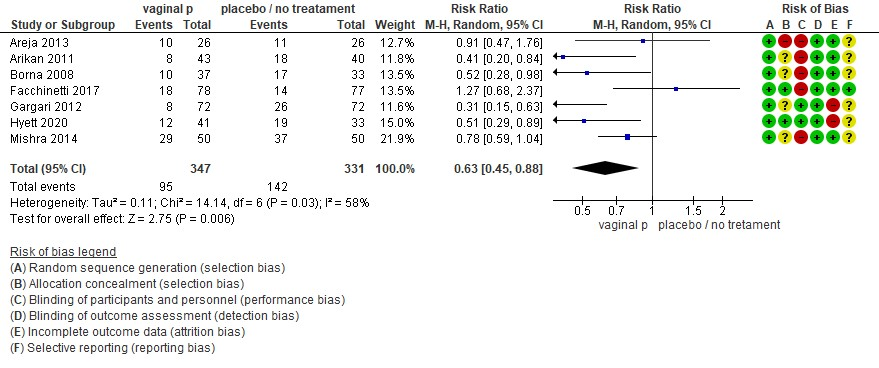

Supplement: S6 Fig — CI, confidence interval; Vaginal P, vaginal progesterone; SD, Standard deviation. (TIF) [file pone.0277563.s006.tif]

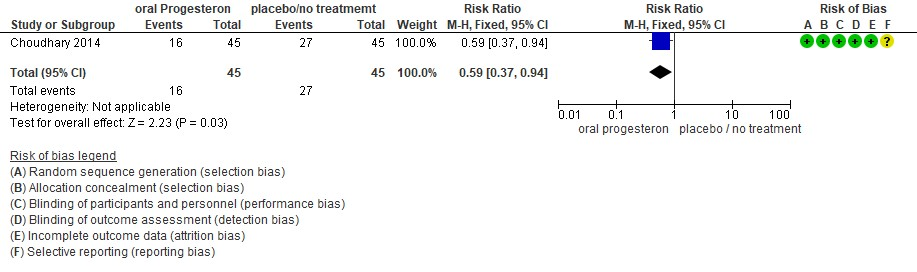

Supplement: S7 Fig — CI, confidence interval; Oral P, oral progesterone; SD, Standard deviation. (TIF) [file pone.0277563.s007.tif]

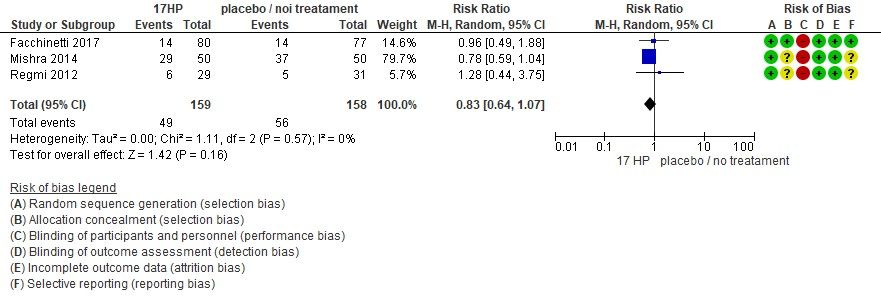

Supplement: S8 Fig — CI, confidence interval; 17-HP, 17-alfa hydroxyprogesterone; SD, Standard deviation. (TIF) [file pone.0277563.s008.tif]

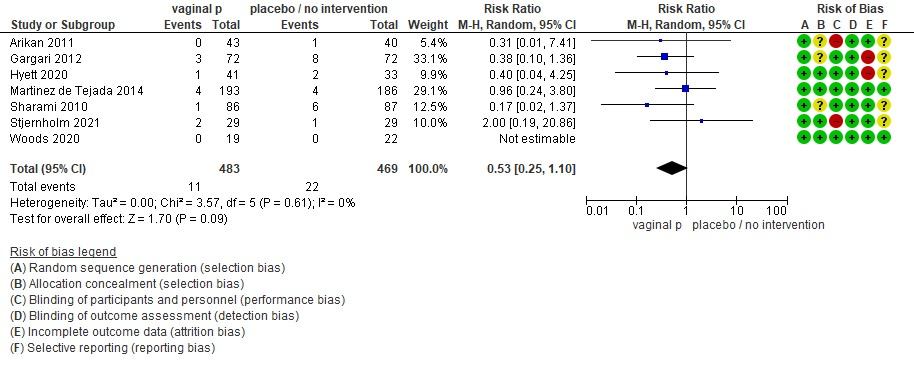

Supplement: S9 Fig — CI, confidence interval; Vaginal P, vaginal progesterone. (TIF) [file pone.0277563.s009.tif]

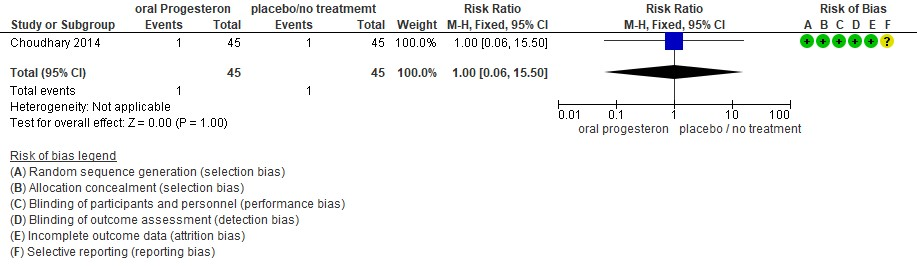

Supplement: S10 Fig — CI, confidence interval; Oral P, oral progesterone. (TIF) [file pone.0277563.s010.tif]

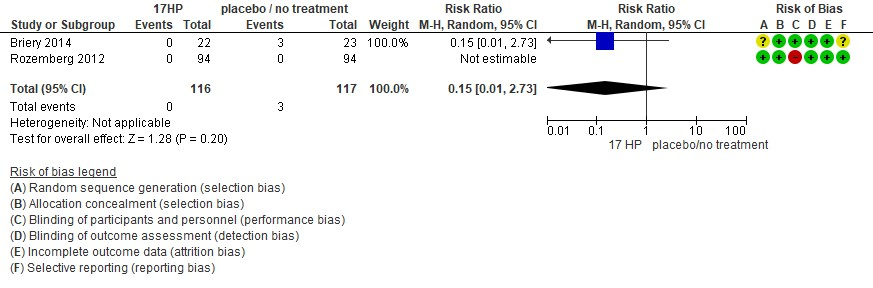

Supplement: S11 Fig — CI, confidence interval; 17-HP, 17-alfa hydroxyprogesterone. (TIF) [file pone.0277563.s011.tif]

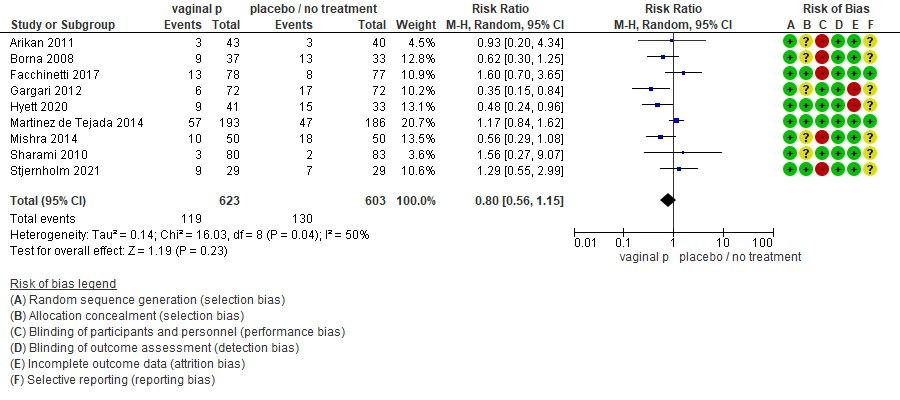

Supplement: S12 Fig — CI, confidence interval; Vaginal P, vaginal progesterone. (TIF) [file pone.0277563.s012.tif]

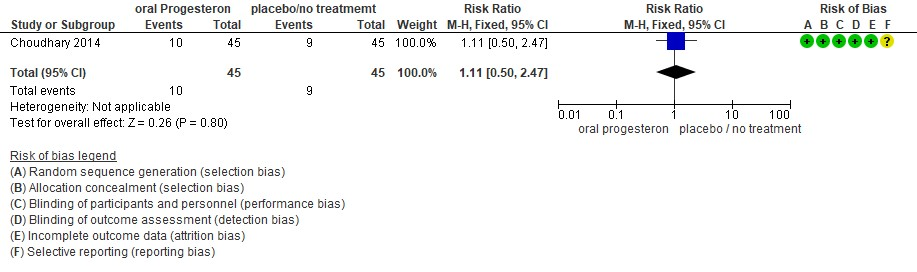

Supplement: S13 Fig — CI, confidence interval; 17-HP, 17-alfa hydroxyprogesterone. (TIF) [file pone.0277563.s013.tif]

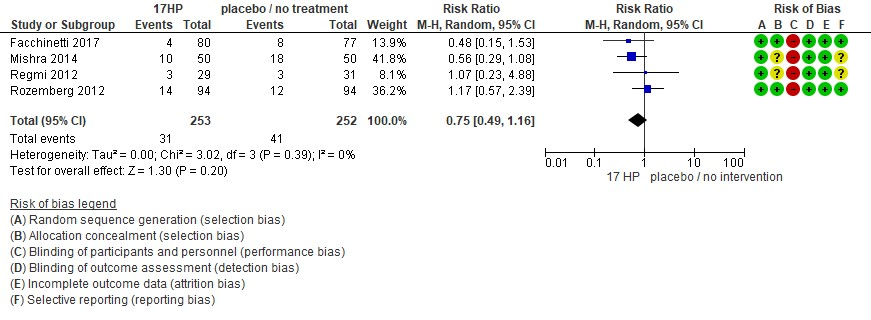

Supplement: S14 Fig — CI, confidence interval; Oral P, oral progesterone. (TIF) [file pone.0277563.s014.tif]

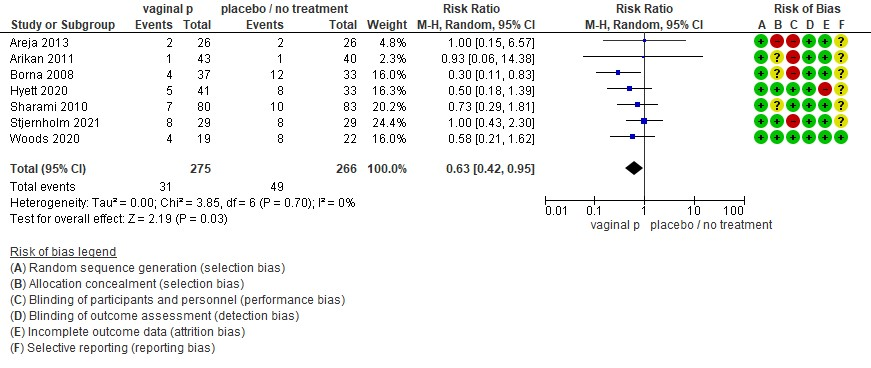

Supplement: S15 Fig — CI, confidence interval; Vaginal P, vaginal progesterone. (TIF) [file pone.0277563.s015.tif]

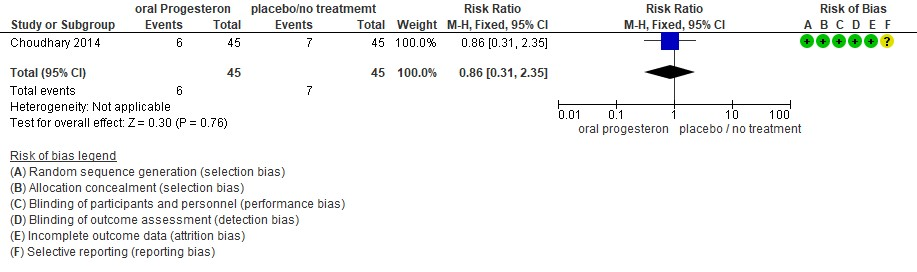

Supplement: S16 Fig — CI, confidence interval; Oral P, oral progesterone. (TIF) [file pone.0277563.s016.tif]

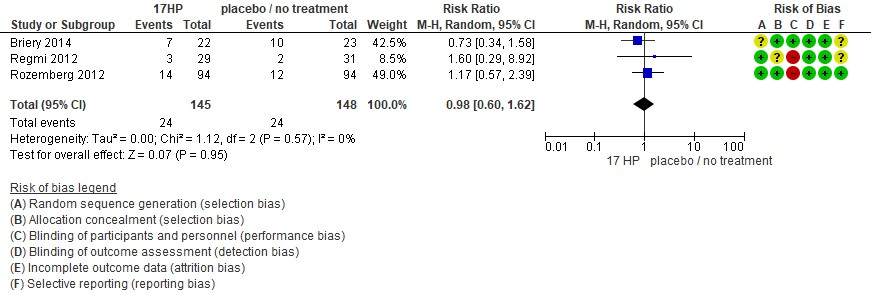

Supplement: S17 Fig — CI, confidence interval; 17-HP, 17-alfa hydroxyprogesterone. (TIF) [file pone.0277563.s017.tif]

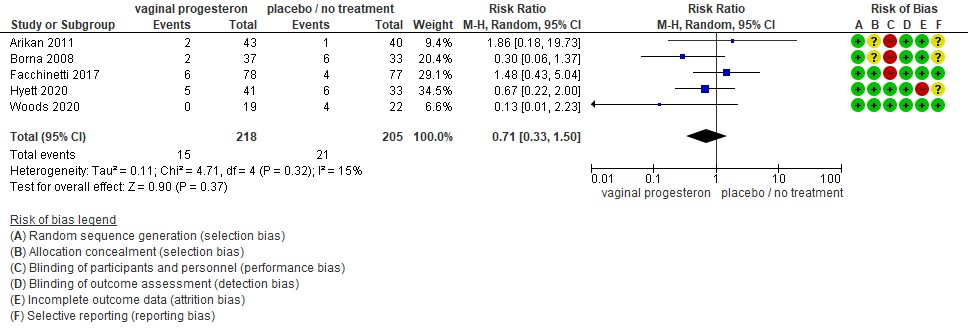

Supplement: S18 Fig — CI, confidence interval; Vaginal P, vaginal progesterone. (TIF) [file pone.0277563.s018.tif]

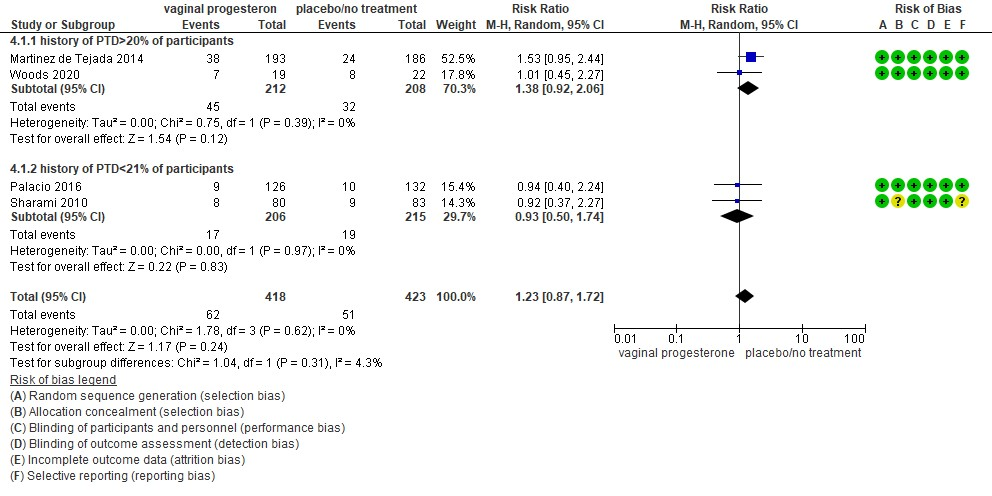

Supplement: S20 Fig — CI, confidence interval; Vaginal P, vaginal progesterone; PTD, Preterm delivery; PTB, Preterm birth. (TIF) [file pone.0277563.s020.tif]

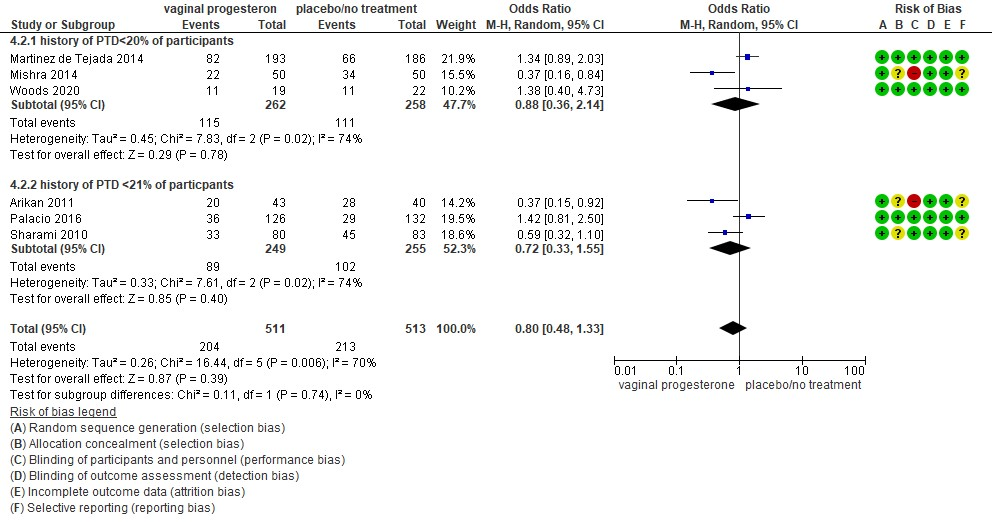

Supplement: S21 Fig — CI, confidence interval; Vaginal P, vaginal progesterone; PTD, Preterm delivery; PTB, Preterm birth. (TIF) [file pone.0277563.s021.tif]
