# Supplementary material for: Progestogens for maintenance tocolysis in symptomatic women. A systematic review and meta-analysis
Source: PLoS One. 2023 Feb 22;18(2):e0277563. doi: 10.1371/journal.pone.0277563 (PMC9946203; doi:10.1371/journal.pone.0277563)
Supplement: S1 Table — Vaginal P, vaginal progesterone; Oral P, oral progesterone; 17-HP, 17-alfa hydroxyprogesterone; PTB, Preterm Labour. (DOCX) [file pone.0277563.s022.docx]

**Table S1**

|  | *PTB and Vaginal P* | *PTB and 17-HP* | *PTB and Oral P* | *Tocolysis and Vaginal P* | *Tocolysis and 17-HP* | *Tocolysis and Oral P* | *Tocolysis and PTB* | *Tocolysis* | *PTB* | *TOT* | Overall |
| --- | --- | --- | --- | --- | --- | --- | --- | --- | --- | --- | --- |
| **MEDLINE** | 12 | 5 | 17 | 16 | 11 | 14 | 12 | 32 | 63 | 182 | **5899** |
| **ClinicalTrials.gov** | 52 | 23 | 10 | 12 | 3 | 3 | 57 | 91 | 1459 | 1710 |  |
| **Cochrane Register of Controlled Trials (CENTRAL)** | 179 | 64 | 40 | 36 | 41 | 14 | 368 | 557 | 2708 | 4007 |  |

Studies selection strategy

Vaginal P, vaginal progesterone; Oral P, oral progesterone; 17-HP, 17-alfa hydroxyprogesterone; PTB,

Preterm Labour
