# Supplementary material for: Progestogens for maintenance tocolysis in symptomatic women. A systematic review and meta-analysis
Source: PLoS One. 2023 Feb 22;18(2):e0277563. doi: 10.1371/journal.pone.0277563 (PMC9946203; doi:10.1371/journal.pone.0277563)
Supplement: S2 Table — CI: Confidence interval; RR: Risk ratio; Oral P: Oral progesterone; Vaginal P: Vaginal progesterone; 17-OH: 17 hydroxyprogesterone. High certainty: We are very confident that the true effect lies close to that of the estimate of the effect. Moderate certainty: We are moderately confident in the effect estimate: The true effect is likely to be close to the estimate of the effect, but there is a possibility that it is substantially different. Low certainty: Our confidence in the effect estimate is limited: The true effect may be substantially different from the estimate of the effect. Very low certainty: We have very little confidence in the effect estimate: The true effect is likely to be substantially different from the estimate of effect. Explanations: a. downgraded two levels for imprecision: very few events. (DOCX) [file pone.0277563.s023.docx]

| **Summary of findings:** | | | | | | | | | | | |
| --- | --- | --- | --- | --- | --- | --- | --- | --- | --- | --- | --- |
| **Vaginal P compared to placebo/no treatment for preterm birth** | | | | | | | | | | | |
| **Patient or population:** participants with singleton gestations that remained undelivered after an episode of preterm labor  **Setting:** outpatients  **Intervention:** vaginal P  **Comparison:** placebo/no treatment | | | | | | | | | | | |
| Outcomes | **Anticipated absolute effects^*^** (95% CI) | | | | Relative effect (95% CI) | | № of participants (studies) | | Certainty of the evidence (GRADE) | | Comments |
|  | **Risk with placebo/no treatment** | | **Risk with vaginal P** | |  |  |  |  |  |  |  |
| PTB < 34 weeks | 128 per 1.000 | | **155 per 1.000** (116 to 206) | | **RR 1.21** (0.91 to 1.61) | | 1106 (7 RCTs) | | ⨁⨁⨁◯ Moderate^a^ | |  |
| PTB <37 weeks | 385 per 1.000 | | **366 per 1.000** (277 to 485) | | **RR 0.95** (0.72 to 1.26) | | 1231 (8 RCTs) | | ⨁⨁⨁⨁ High | |  |
| **17-HP compared to placebo/no treatment for preterm birth** | | | | | | | | | | | |
| **Patient or population: participants with singleton gestations that remained undelivered after an episode of preterm labor**  **Setting: outpatients**  **Intervention: 17-HP**  **Comparison: placebo/no treatment** | | | | | | | | | | | |
| Outcomes | **Anticipated absolute effects^*^** (95% CI) | | | | Relative effect (95% CI) | | № of participants  (studies) | | Certainty of the evidence (GRADE) | Comments | |
|  | **Risk with placebo/no treatment** | | **Risk with 17-HP** | |  |  |  |  |  |  |  |
| PTB < 34 weeks | 219 per 1.000 | | **158 per 1.000** (118 to 208) | | **RR 0.72** (0.54 to 0.95) | | 450 (4 RCTs) | | ⨁⨁⨁◯ MODERATE ^a^ |  | |
| PTB <37 weeks | 397 per 1.000 | | **342 per 1.000** (238 to 481) | | **RR 0.86** (0.60 to 1.21) | | 450 (4 RCTs) | | ⨁⨁⨁◯ MODERATE ^a,^ |  | |
| **Oral P compared to placebo/no treatment for preterm birth** | | | | | | | | | | | |
| **Patient or population: participants with singleton gestations that remained undelivered after an episode of preterm labor**  **Setting: outpatients**  **Intervention: oral P**  **Comparison: placebo/no treatment** | | | | | | | | | | | |
| Outcomes | | **Anticipated absolute effects^*^** (95% CI) | | | Relative effect (95% CI) | № of participants  (studies) | | Certainty of the evidence (GRADE) | | Comments | |
|  |  | **Risk with placebo/no treatment** | | **Risk with oral P** |  |  |  |  |  |  |  |
| PTB < 34 weeks | | 200 per 1.000 | | **178 per 1.000** (76 to 420) | **RR 0.89** (0.38 to 2.10) | 90 (1 RCT) | | ⨁⨁◯◯ LOW ^a^ | |  | |
| PTB <37 weeks | | 578 per 1.000 | | **335 per 1.000** (208 to 537) | **RR 0.58** (0.36 to 0.93) | 90 (1 RCT) | | ⨁⨁◯◯ LOW ^a^ | |  | |
