# Supplementary material for: Progestogens for maintenance tocolysis in symptomatic women. A systematic review and meta-analysis
Source: PLoS One. 2023 Feb 22;18(2):e0277563. doi: 10.1371/journal.pone.0277563 (PMC9946203; doi:10.1371/journal.pone.0277563)
Supplement: Attachment — Submitted filename: Response to Reviewers 2.docx [file pone.0277563.s025.docx]

**Specifically, how can the Briery 2014 report have "not substantially different risk of bias" when the enrollment of the entire study did not reach a priori sample size requirements because the fellow graduated when compared to the other studies? In the Briery study, 45 singleton pregnancies were randomized after successful tocolysis; however, the methods stated, "sample size calculation suggested that 80 treated pregnancies (40 in each group) would yield a sufficient number to have an 80% power of detecting a 20% reduction in delivery before 37 weeks." That is, this study did not enroll a prior sample size needed for a primary outcome of <37 weeks.

The small sample is less weighted but carries the bulk of "benefit" of 17OHP-C on a secondary outcome of an underpowered study. This is a key element in the analysis as this drives the final conclusions for 17OHP-C.**

We thank the Reviewer for the comment.

We reported that studies, including Briery 2014, have "not substantially different risk of bias" because the sample size and the ability or not of the researchers to achieve the predetermined sample size to have an adequate power does not introduce bias, according to the Cochrane Criteria, the most accepted and universally used tool to assess RoB of studies included in systematic reviews. As a matter of fact, Briery 2014 study was included in all the previous metanalyses ^( 9; 11; 40^). The small sample size however impacts on the precision of the estimate of this study, not on risk of bias. Therefore, we accept your comment and added to the text that the precision of the estimate could be reduced, due to the small number of events (see text changes in the discussion section).

Finally, within the perspective of a SR and meta-analysis, it does not matter whether the primary studies defined an outcome as primary or secondary. All the data are pooled and studies possibly underpowered to detect a difference because the outcome was defined as secondary, will contribute less to the overall estimate.

Text changes:

**Line 276**: “It should be underlined that the relatively small number of events impacts on the precision of the estimate.”
